# Alfalfa Carbon and Nitrogen Sequestration Patterns and Effects of Temperature and Precipitation in Three Agro-Pastoral Ecotones of Northern China

**DOI:** 10.1371/journal.pone.0050544

**Published:** 2012-11-28

**Authors:** Shujuan Chang, Nan Liu, Xiaoya Wang, Yingjun Zhang, Yue Xie

**Affiliations:** 1 Institute of Grassland Science, China Agricultural University, Beijing, People’s Republic of China; 2 Inner Mongolia Rangeland Survey & Design Institute, Hohhot, Inner Mongolia, People’s Republic of China; DOE Pacific Northwest National Laboratory, United States of America

## Abstract

Alfalfa (*Medicago sativa* L.) is a primary and widely cultivated forage crop in China. As a perennial leguminous grass, continuous planted alfalfa may influence carbon and nitrogen sequestration in soils. We evaluated the effect of alfalfa, planted for different lengths of time, and temperature and precipitation on soil organic carbon (SOC) and total nitrogen (TN) contents, and estimated soil SOC and TN inventories from 0–60 cm in three agro-pastoral ecotones of northern China. Alfalfa SOC and TN storage patterns were significantly different with increasing soil depths between the three regions of northern China. Continuous alfalfa grassland planted had a positive effect on accumulation of both SOC and TN in the Northwest region, whereas SOC storage peaked 6–7 years after planting in the Northeast and North region. Moreover, relatively higher TN storage appeared 7 years after planting in the Northeast and North regions. This study controlled as many factors as possible, but we caution that such temporal inferences could be artifacts of site selection. The regression analysis indicated that SOC and TN accumulation was mainly dependent on temperature (≥10°C of effective total accumulated temperature) in the North region. Precipitation in the growing season was the main limiting factor for SOC storage in the Northwest region and TN accumulation in the North regions. Therefore, the different climate factors affecting SOC and TN sequestration in alfalfa occurred at a regional scale.

## Introduction

Soil is a vital component of the global carbon cycle [1]. Soil organic carbon (SOC) is crucially significant in sustaining soil quality, crop production and environmental quality [2]. A sustainable soil C pool will mitigate increasing CO_2_ levels in the atmosphere, and consequently have a substantial influence on the C content of the atmosphere [3]–[5]. How to enhance the potential of the soil C pool to sequester soil carbon, and thus alleviate the rate of increase of CO_2_ concentrations in the atmosphere, has become an important research area globally.

Carbon sequestration is a biotic process. Carbon fixed by photosynthesis is the primary source of SOC, with the organic litter being decomposed and converted into soil organic matter [6]. In grassland ecosystems, more than 90% of the organic carbon is present in roots and soils [7]. Many studies have shown that changes in land-use are inevitably accompanied by variations in soil carbon storage [8]–[10]. A change of land-use from native vegetation to agriculture resulted in sharp declines in soil organic matter [11], and the conversion of annually cultivated land to forage grasses has the potential to increase C and N sequestration [12], [13]. However soil carbon inventories and turnover rates are also influenced by climate, vegetation, parent material, topography, time, and the fundamental state factors of soil formation [14], [15], and so soils developed under different vegetation types and climates may have different modes of soil organic matter stabilization [16]. Climate change and their interaction with vegetation have been acknowledged as one of the main driving forces of SOC variations [17]. Many authors, studying very different environments, have found that the SOC content is directly proportional to the mean annual precipitation and inversely proportional to the mean annual temperature [17]–[23]. The selection and operation of appropriate land management measures for mitigating atmospheric carbon concentrations will ultimately depend on knowledge of the carbon sequestration potential under various types of land-use in different ecosystems and biomes. At the same time, we need to consider potential compromises that may arise from changes in land-use with respect to food and fiber production that support global socio-economic activities [10]. Knowledge of soil carbon sequestration by particular plants in different spatial arrangements and temporal changes in that sequestration will enable better use of the land and achievement of optimum ecological and economic benefits.

Alfalfa (*Medicago sativa* L.) is a perennial leguminous grass that is widely planted through the world. In China alfalfa has been grown for more than 2000 years, primarily as a cultivated forage crop, and is widely distributed in northern regions. In 2009, it was estimated that the alfalfa-planted area in China reached 2.80 million hectares [24]. As a principal crop in legume-cereal rotations, alfalfa plays an important ecological and economic role in the agro-pastoral ecotones of China. Alfalfa is also a primary species used to restore vegetation in the National Western Development Program [25]. In China, many studies on alfalfa grassland converted by cropland showed that this land use change in favor for soil carbon sequestration, but most of those study sites were form the semiarid Loess Plateau, Northwest of China[13], [25]–[26]. Investigating soil carbon dynamics under continuously planted alfalfa will also contribute to the taking of suitable measures to alleviate the rate of increase of CO_2_ levels in the atmosphere.

The objective of the present study was to investigate at large regional scale spatial and temporal changes in the soil carbon pool under long-term plantings of alfalfa converted from annually cultivated land. To explain the differences in dynamics of the soil C and TN storage of alfalfa grassland from three main cultivation regions, temperature and precipitation data (China Meteorological Date sharing Service System: http://cdc.cma.gov.cn/) were used in stepwise multiple regression analysis to establish which factors may be responsible for the observed differences. Based on the above content, three questions are investigated. First, what are the differences among the three regions in carbon and nitrogen sequestration in soil? Second, which are the primary climatic factors in this process? And third, whether there is a threshold in the soil C and TN storage under alfalfa grassland?

## Materials and Methods

### Study site

**Figure 1:**
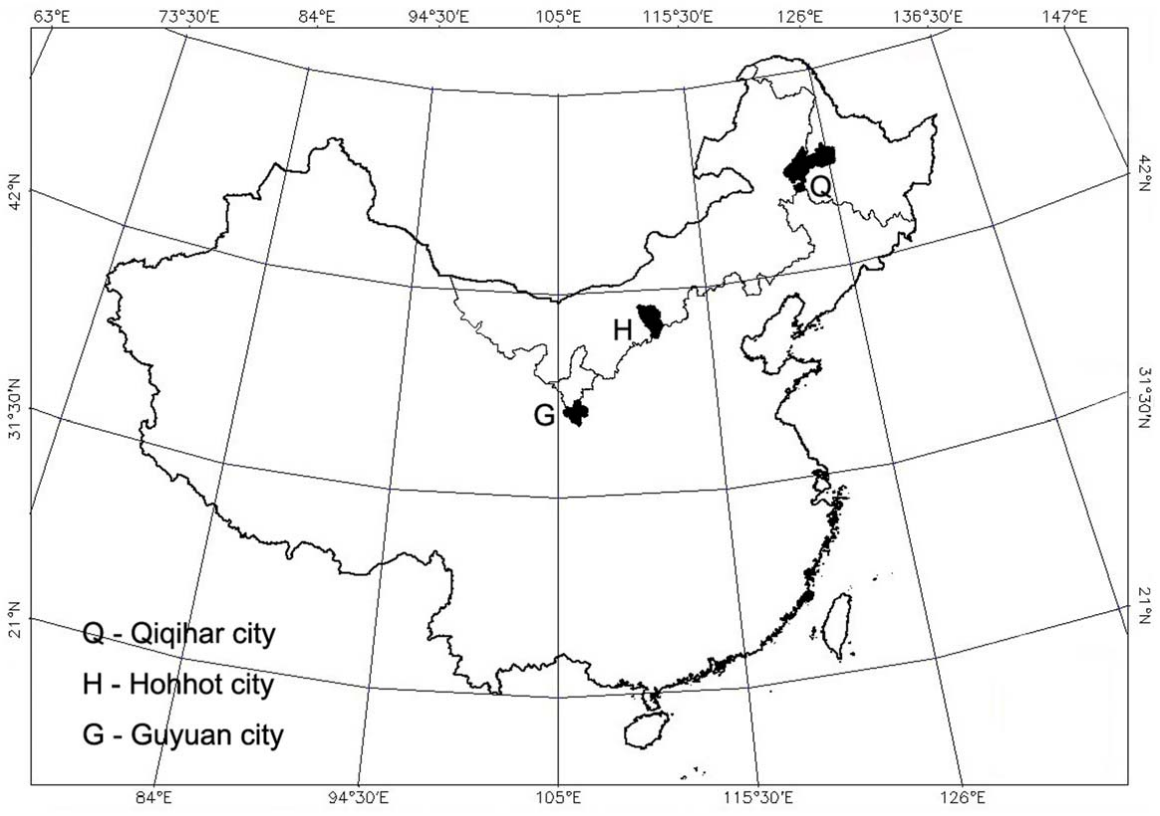
Location of the study areas in China.

The study area is shown in Fig.1. It covers the main cultivation regions of alfalfa grassland from east to west in the agro-pastoral ecotone of the north of China. Three sites were sampled along this gradient, with each region having different climate and soil types, but a similar cultivation history. One site (the Northeast region) was located near city of Qiqihar in the northeast of China, on a sandy black loam. The second site, located near city of Hohhot, in the Inner Mongolia Autonomous Region of China (the North region), was established on a light chestnut soil. The third site was located near city of Guyuan, which is on the Loess Plateau, in Ningxia province of northwest China (the Northwest region), on a light black loessial soil and a cultivated loessial soil.

**Figure 2:**
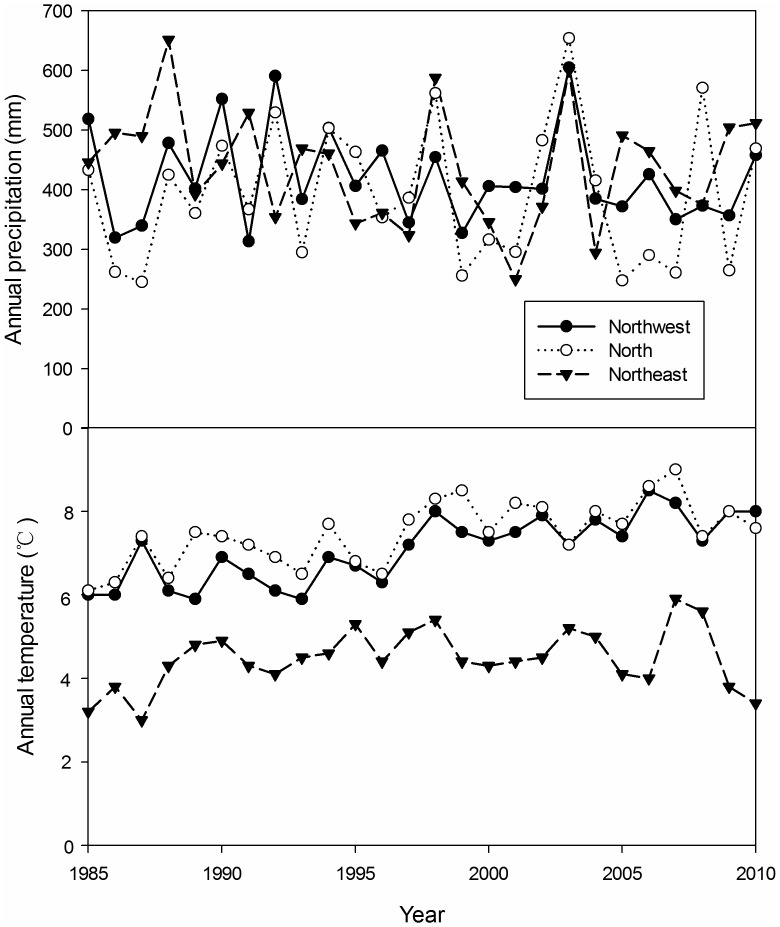
Annual precipitation (mm) and annual temperature (°C) in the three regions from 1985 to 2010. The data from China Meteorological Date sharing Service System: http://cdc.cma.gov.cn/.

The climate of the Northeast region is typified by a windy dry spring and cold winter with an average annual temperature of 4.5°C and mean annual precipitation of 437 mm according to the statistical data from 1985 to 2010. The climate of the North region has an average annual temperature of 7.5°C, and mean annual precipitation of 392 mm. The Northwest region has a moderate semiarid climate, that with an average annual temperature of 7.09°C, and mean annual precipitation of 421 mm (Fig.2).

Samples were selected from 17 fields in 2010, comprising six alfalfa grasslands from the Northeast region, six from the North region and five from the Northwest region with the samples from each region being from fields where alfalfa had been growing for different numbers of years (Table 1). In northeast China, the six alfalfa grasslands in Qiqihar were planted in different years in an area belonging to the Institute of Animal Science in Heilongjiang province. The six alfalfa grasslands from Hohhot of the North region, all planted in different years, belong to the Shaerqin forage resource field scientific observation station of the Grassland Research, Institute of the Chinese Academy of Agricultural Sciences. The five alfalfa grasslands from Guyuan in the Northwest region, all planted in different years, are mowed grassland that was planted by local farmers. All of the grasslands are cut two or three times per year, and followed maize crops.

**Table 1:** Details of soil samples from alfalfa grasslands growing for different numbers of years in the three regions.

(Northeast Region) Qiqihar		(North Region) Hohhot		(Northwest Region) Guyuan	
Ab.	No. of growth years	Ab.	No. of growth years	Ab.	No. of growth years
QM10	10-yr alfalfa grassland	HM9	9-yr alfalfa grassland	GM17	17-yr alfalfa grassland
QM8	8-yr alfalfa grassland	HM7	7-yr alfalfa grassland	GM10	10-yr alfalfa grassland
QM6	6-yr alfalfa grassland	HM5	5-yr alfalfa grassland	GM7	7-yr alfalfa grassland
QM4	4-yr alfalfa grassland	HM4	4-yr alfalfa grassland	GM4	4-yr alfalfa grassland
QM3	3-yr alfalfa grassland	HM2	2-yr alfalfa grassland	GM1	1-yr alfalfa grassland
QM2	2-yr alfalfa grassland	HM1	1-yr alfalfa grassland		
Latitude	47°15′43.70″		40°36′11.05″		36°11′41.32″
longitude	123°40′32.30″		111°45′24.45″		106°24′35.64″

### Soil Sampling and Preparation

Soil samples were taken at the end of the grassland growth period on September 5, 2010 in the Northeast region, September 9th in the North and the 14th in the Northwest, respectively. Five sub-samples were randomly collected using a coring tube (diameter 5 cm) and mixed as one composite sample. The soil sampling was replicated five times for each field. Soil samples were collected from depths of 0–5 cm, 5–10 cm, 10–20 cm, 20–30 cm, 30–45 cm, and 45–60 cm. After air-drying, the soils were placed in self-sealing bags and returned to the laboratory for soil organic C (SOC) and total N (TN) analyses. Another three soil samples were collected from the same depth increments in the same fields using a cutting ring (volume of 100 cm^3^) for measurement of the soil bulk density.

### Measurements and Soil Analyses

Soil samples from the 17 fields were analyzed for soil bulk density, SOC, and TN. The soil samples for SOC and TN analyses were sieved through a 1 mm sieve. SOC concentrations were determined by the Walkley-Black method [27]. TN concentrations were determined using the Kjeldahl wet digestion procedure [28], using a 2300 Kjeltec Analyzer Unit (FOSS, Sweden). SOC and TN concentrations were multiplied by the soil bulk density to give the SOC and TN mass per unit area per 1 cm depth for the six sampled soil layers.

### Statistical Methods

Regression analyses were used to investigate the relationship between SOC or TN storage and growing years in the three regions. Multiple stepwise regression was used to investigate the correlation of SOC and TN sequestration with ≥10°C of the effective total accumulated temperature, T_e_, and total precipitation (mm) in the growing reason (April to October), P_g_. The temperature and precipitation data were from China Meteorological Date sharing Service System: http://cdc.cma.gov.cn/. The analysis was carried out using SPSS (SPSS, 2000).

## Results

### Soil Bulk Density, SOC and TN Storage Changes

**Figure 3:**
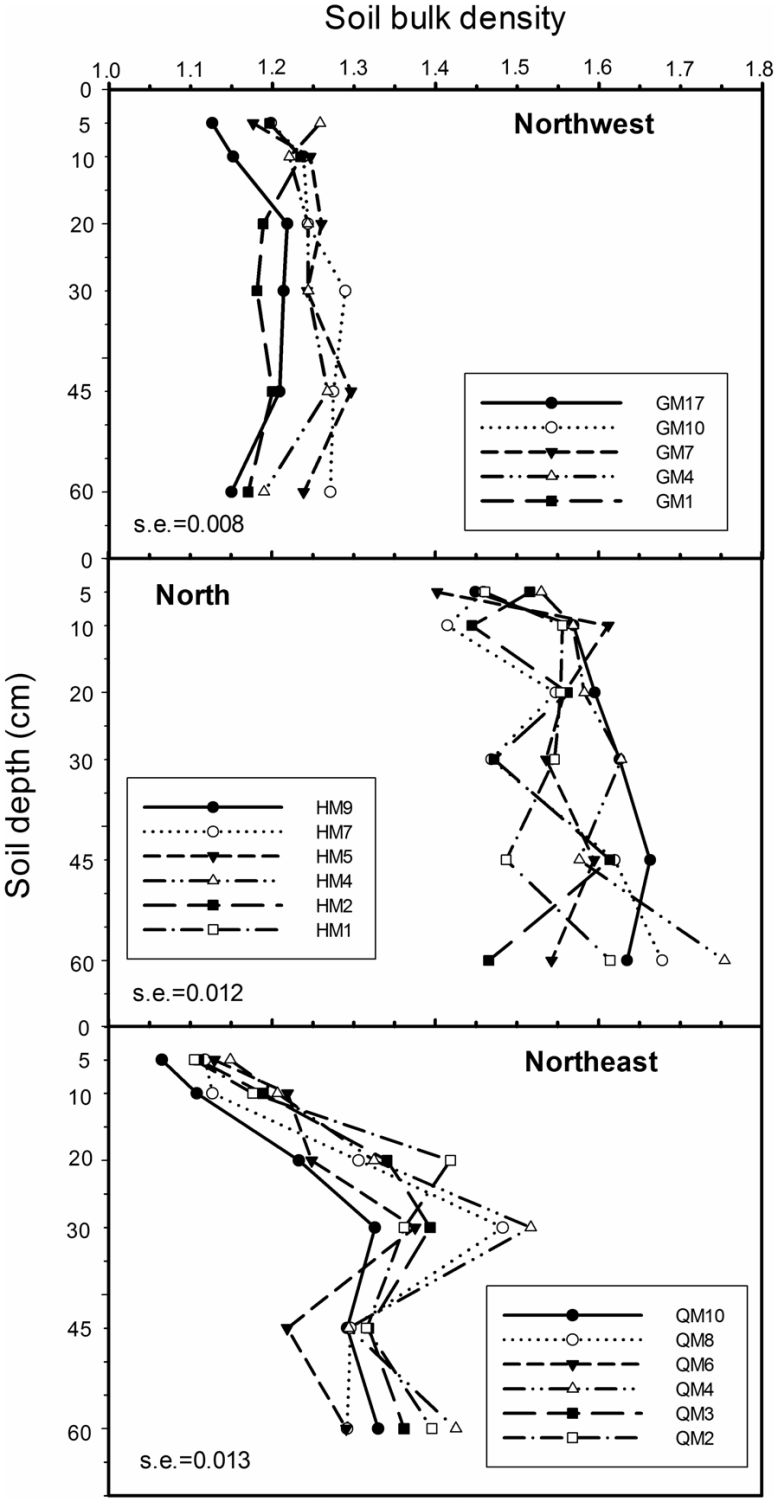
Mean soil bulk density in different soil layers from the three regions. The value of standard error (s.e.) for each region was stated in the graph (n = 90 for Northwest, n = 108 for North and Northeast region).

There were no changes in bulk density among soil layers in the Northwest region. Moreover, there was a trend of increasing bulk density with time since planting, except for the 17-year-old field. In the Northeast region, an increase in bulk density with the increase of soil depths appeared in the top 30 cm soil layer, and then declined. The soil bulk density was in the order of North>Northeast>Northwest (Fig. 3).

**Figure 4:**
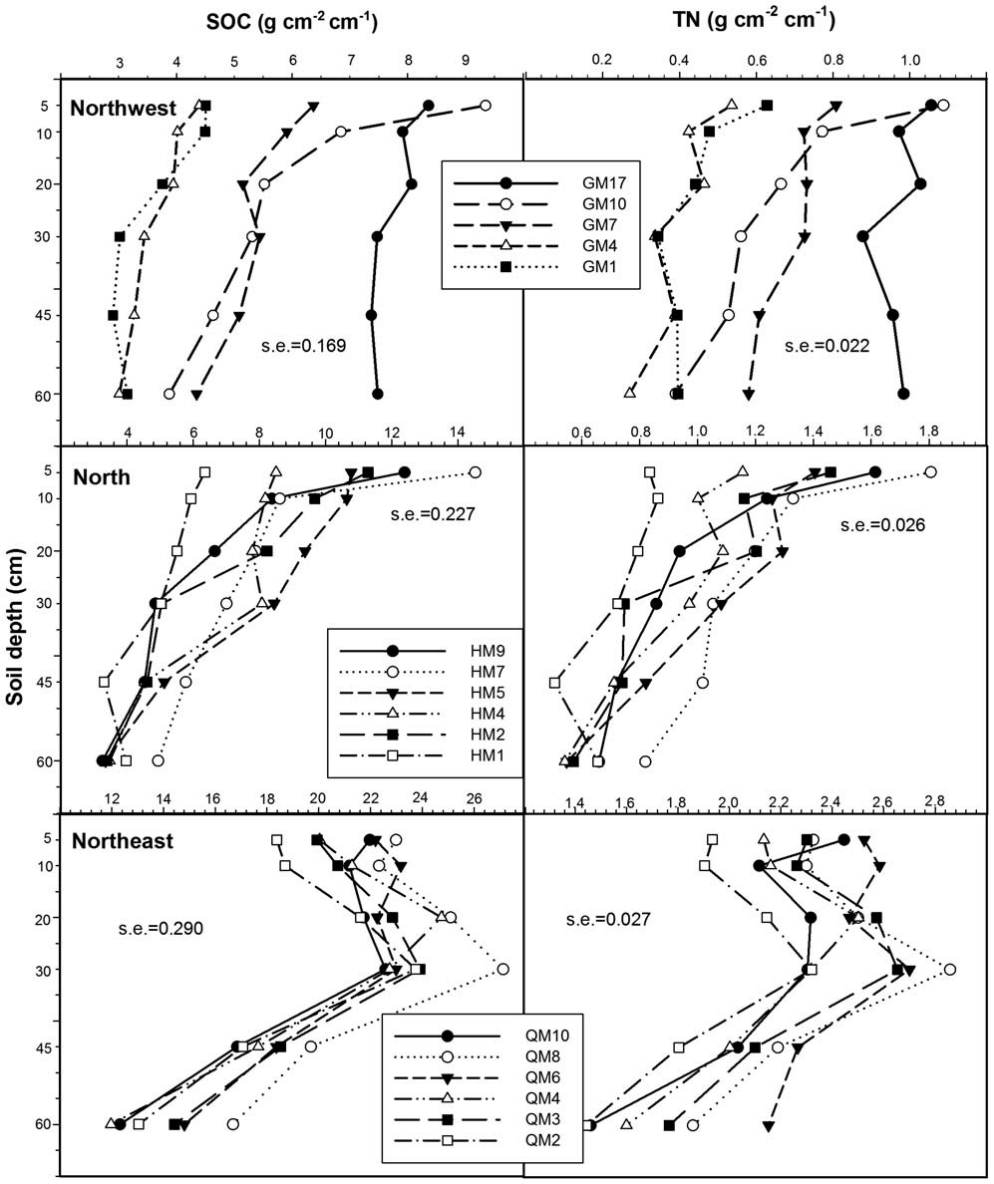
Mean SOC and TN storage in different soil layers from the three regions. The value of standard error (s.e.) for each region was stated in the graph (n = 150 for Northwest, n = 180 for North and Northeast region).

**Figure 5:**
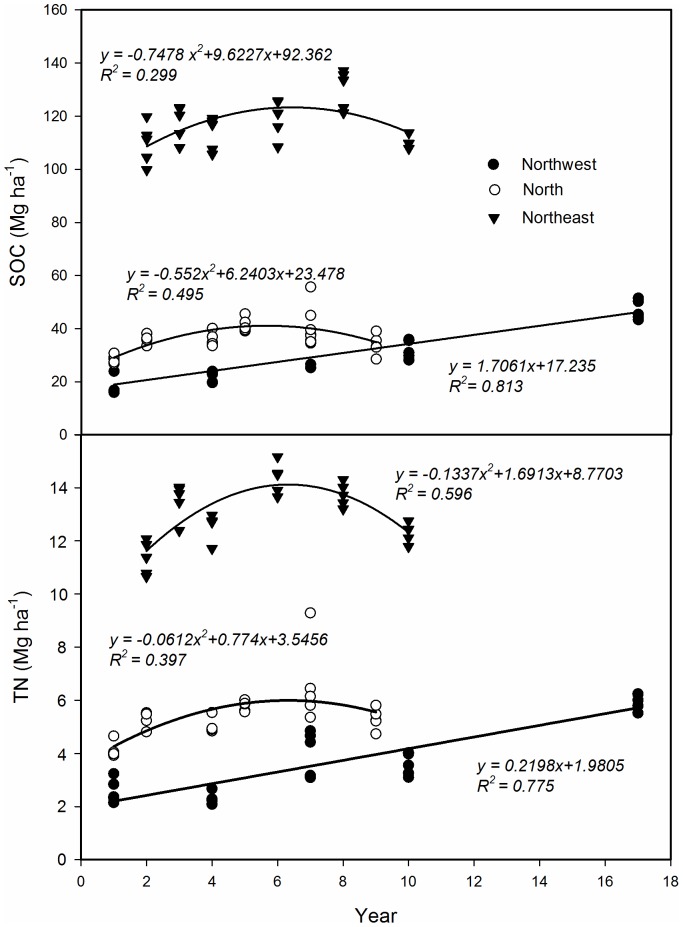
The relationship of soil organic carbon (SOC) and total nitrogen (TN) storage with years of growing alfalfa in North, Northeast and Northwest region of China.

In the North region, SOC and TN storage was far higher in surface soil than in sub-layer soil, and decreased with increasing of soil depth. Compared with the sharp decrease of SOC and TN with the increase of soil depth in the North, SOC and TN storage had a relative slight decrease in the Northwest. In the top 30 cm, SOC and TN storage was increased with increasing of soil depth, but decrease from 30 to 60 cm soil layer in the Northeast. The SOC and TN storage were in the order of Northeast>North>Northwest (Fig. 4).The regression model (Fig. 5) showed the relationship between the growing years and SOC and TN storage on 0–60 cm layers in alfalfa grassland. In the North region, SOC storage was increased with increasing of growing years, and reached the maximal storage after growing six years (41.11 Mg/ha), then begin to decline (n = 30, *P*<0.05; *R^2^* = 0.495 ). Similarly with TN storage, the maximal storage was appeared in 7th years of 5.99 Mg/ha in the North region (n = 30, *P*<0.05; *R* ^2^ = 0.397). In the Northeast region, the peak of SOC and TN storage was appeared in 7th years of 123.32 and 14.12 Mg/ha (n = 30, *P*<0.05; *R^2^* = 0.299 in SOC and 0.596 in TN). Soil carbon and nitrogen storage in the Northwest region had a significantly positive linear correlation relationship with the growing years (n = 25, *P*<0.05; *R^2^* = 0.813 in SOC and 0.775 in TN).

### The Regression Model of SOC and TN Storage in Relation to Temperature and Precipitation

**Figure 6:**
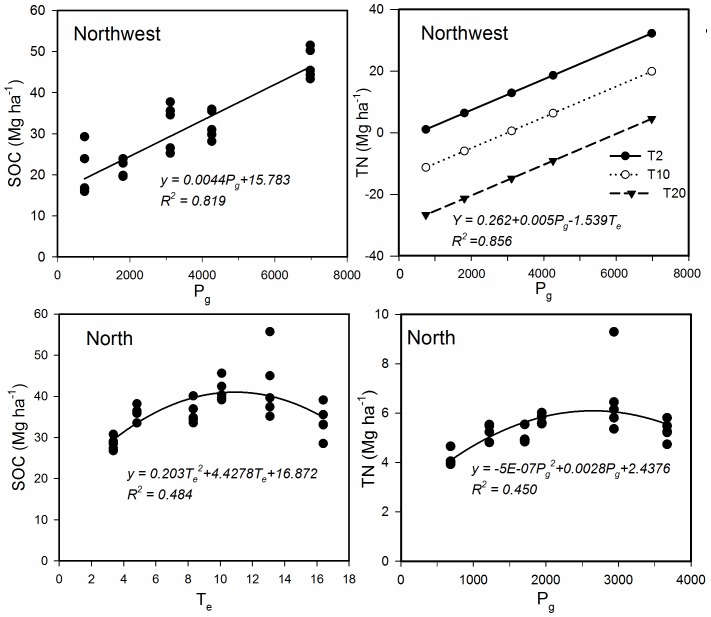
Relationships between SOC or TN (0–60cm) and ≥10°C of the effective total accumulated temperature (T_e_) or total precipitation (mm) in the growing reason (P_g_) in the North and Northwest region.

In the Northeast region, the stepwise regression analysis (Fig. 6) showed that SOC and TN storage cannot be quantitatively described by a combination of T_e_ or P_g_. In the Northwest region, SOC storage increased with increasing T_e_ (n = 25, *R^2^* = 0.819, *P*<0.001), indicating that approximately 81.9% of the variability in SOC can be explained by variation in T_e_. But TN storage can be quantitatively described by a linear combination of both T_e_ and P_g_ (n = 25, *R^2^* = 0.856, *P*<0.001). Similarly, in the North region, SOC storage was correlated with T_e_ in the 0–60 cm layer (n = 30, *R^2^* = 0.484, *P*<0.05), but TN storage was weakly correlated with P_g_ in the 0–60 cm layer (n = 30, *R^2^* = 0.450, *P* = 0.05). However, the correlation coefficients are all significant. Meanwhile, the linear regression analysis indicates that soil C in the 0–60 cm layer is more sensitive to changes in temperature than TN storage in the North region soils. But the TN (0–60 cm depths) sequestration is more sensitive to changes in precipitation than effective accumulated temperature in the Northwest region.

## Discussion

### Dynamics of SOC and TN

SOC and TN storage patterns among the three regions were very different in alfalfa grassland, with the Northeast having the highest storage and the Northwest the lowest. Different background conditions together with differences in soil type and climate may cause the diversity of settings on SOC and TN storage in three regions. Soil carbon and nitrogen sequestration are long-term biotic processes that are affected by climate, plant species abundance and productivity, litter decomposition, soil structure, and agricultural practices [29]–[34]. Changes in SOC often lead to differences in soil total N, because organic forms generally account for more than 95% of the soil N [35].

With respect to differences in the length of time that alfalfa has been planted, the amount of carbon increased with an increase in the planting time, representing a considerable accumulation of SOC in these soils following conversion of annual crops to a continuous alfalfa system. But the increase trends with the increase in planting time had a threshold in the Northeast and North regions. The Northwest region had less carbon sequestration than the other regions. Meanwhile, in the Northwest region, soil bulk density was lower than the other region, with no significant difference between the six depths. The relative lower soil bulk density led to the lower capability on fertilizer and water conservation in the soil arable layer. This means that the conversion of annual cropland to the continuous alfalfa grassland in this poor soil may have greater potential on the accumulation of soil organic carbon and nitrogen. In the Northeast region, soil bulk density on 20–30 cm layers was significant higher than other layers, that caused by the plow pan profile on this layer. Long-term cultivation of a single crop in company with surface tillage will lead to thickening of the plow pan profile, soil compaction and bulk density increased [36]. Fluctuations of the SOC content in the Northeast region are gentler than in the other regions, indicated by smaller differences with increasing soil depth. In the North region, the SOC and TN contents in the 30–60 cm layer are significantly lower than in the 0–30 cm layer. This is possibly because the soil in the North region is a chestnut soil that has a calcic horizon at 30–60 cm [37]. The tight structure of calcic horizons influences root growth.

In recent years, there has been considerable research into changes in soil properties when annual cropland is converted to perennial forage. Such conversions have been shown to increase soil carbon and nitrogen contents [12]–[13], [25]. However, Jiang et al. [26] considered that removal of large quantities of forage from alfalfa grassland systems through mowing, which is the continuous alfalfa system used in Northwest China, can exhaust soil water leading to severe soil dryness, and deplete SOC and soil nutrients in the 0–100 cm soil layer.

How to identify changes in organic matter is crucial in defining sustainable agricultural practices [38]. Changes in SOC storage in the three regions under the continuous alfalfa system are not consistent. In the North and Northeast region, SOC and TN storage changes did not show a consistent trend with the increase in the number of growing years. However, SOC storage in the Northwest region showed significant increases with increasing years of growing alfalfa. This is consistent with other research findings [13], [39]–[40], which found considerable accumulation of SOC in soils where the land use had changed from annual crops to perennial grasses.

We used a chronosequence approach (space-for-time) to study changes of SOC and TN storage in alfalfa with different growing time. The increasing trends of increasing planting time had a threshold in the Northeast and North regions, so temporal results (e.g., strange decline in SOC after 6 years in Figure 5) are potentially confounded with site selection problems, such as spatial heterogeneity. Although we controlled for many factors, we caution that such temporal inferences could be artifacts of site selection. Walker et al. [41] suggested that if the date of the initial disturbance and subsequent history of the site are known, chronosequences provide the opportunity to study ecological processes over time periods that are longer than direct observation would permit. With careful site selection the chronosequence approach is thus an effective research method for evaluating.

### The Relationship between Soil C and Temperature and Precipitation

Changes in temperature and precipitation may affect the soil and vegetation, with the soil affecting vegetation through its influence on water availability, elemental cycling and the soil temperature regime at an ecosystem level [42]. How to balance the effects between the temperature and the precipitation on SOC content determines what climatic factors in a specific area will be limited or decision of the soil carbon sequestration mode. In Canada, Fantappiè et al. [23] indicated that the most remarkable climate effect was that high air temperatures corresponded to low SOC values, probably because of SOC mineralization. High precipitation instead, when considered alone, had a positive effect on SOC content, but their interaction with temperature seemed to have more effect on SOC variations. In the Northeast region the accumulation of SOC and TN in the 0–60 cm layer does not agree with a regression on the regional temperature or precipitation. Precipitation is the main limiting factor in the Northwest region and the North region on TN sequestration. However, in the North region, the accumulation of SOC in the 0–60 cm layer is mainly dependent on the regional temperature. Temperature and precipitation together determine the changes in TN storage in the Northwest region. This suggests that other factors, like cultivation history, the type of cultivation, and soil properties, have more contribution to the changes of soil C and N in Northeast region. Nitrogen sequestration in soil is more sensitive to changes in precipitation. Jenny [15] indicated a strong inter-dependence between climate and soil quality. Water is the key factor limiting plant growth in arid and semi-arid regions [43], and it also determines vegetation composition and distribution patterns [44], [45].

### Conclusion

Alfalfa SOC and TN storage patterns with increasing soil depths were significantly different between the three regions of northern China. Continuous alfalfa grassland planted had effective accumulation of both SOC and TN in the Northwest region. The peak SOC and TN storage were appeared in 6 and 7 years after alfalfa established in the North region, and both 7 years in the Northeast region. Temperature had effect on SOC storage in the North regions, whereas precipitation had more contribution to the SOC storage in the Northwest region and TN storage in the North region. Temperature and precipitation together determine the changes in TN reserves in the Northwest region. In short, SOC and TN storage has close relationship with the changes of temperature and precipitation, especially in the Northwest region.
